# Editorial: Enhancing plant resilience to abiotic stress through biochar application

**DOI:** 10.3389/fpls.2026.1859358

**Published:** 2026-05-07

**Authors:** Nitika Kapoor, Geetika Sirhindi

**Affiliations:** 1 Department of Botanical and Environmental Sciences, Guru Nanak Dev University, Amritsar, Punjab, India; 2 Department of Botany, Punjabi University, Patiala, Punjab, India

**Keywords:** abiotic stress, agriculture, biochar, growth, physiology, resilience

Abiotic stresses such as salinity, drought, and heavy metal toxicity are among the most critical factors limiting global agricultural productivity and sustainability (Maurya and Kumar, 2026). These stresses disrupt plant physiological, biochemical, and molecular processes, ultimately leading to reduced crop yield and quality (Ramzan et al., 2026). With the increasing severity of climate change and environmental degradation, there is an urgent need to develop sustainable and efficient strategies to enhance plant resilience. In this context, biochar has emerged as a promising amendment due to its unique physicochemical properties and its ability to improve soil health and plant stress tolerance.

The Research Topic, *“Enhancing Plant Resilience to Abiotic Stress Through Biochar Application”*, presents four original research articles that collectively advance our understanding of biochar-mediated stress mitigation across different crops and environmental conditions. The contributions in this Research Topic highlight not only the individual effectiveness of biochar but also its synergistic interactions with biological agents and signaling molecules.

Salinity stress, a major constraint in arid and semi-arid regions, is addressed in the study by Alotaibi. The author investigated the combined effects of biochar, compost, and arbuscular mycorrhizal fungi (AMF) on maize growth under saline conditions. The findings demonstrate that while individual treatments improved plant performance, combined applications particularly AMF with biochar resulted in significantly greater benefits *viz.* enhanced biomass production, improved chlorophyll content, and increased uptake of essential nutrients such as nitrogen, phosphorus, and potassium. Moreover, the treatments effectively reduced sodium accumulation and improved ionic balance, as reflected in a higher K:Na ratio. The observed improvements were closely associated with enhanced antioxidant enzyme activities and osmolyte accumulation, indicating reduced oxidative stress. This study underscores the importance of integrated approaches in which biochar acts synergistically with microbial inoculants to enhance plant tolerance to salinity.

Drought stress, another major abiotic constraint, is explored in the work of Zhang et al. This study evaluated the impact of walnut shell-derived biochar on tomato plants under varying water availability conditions. Biochar application significantly improved root architecture, including increased primary root length and lateral root density, thereby enhancing water uptake efficiency. Additionally, biochar-treated plants exhibited improved biomass accumulation and moderated levels of stress-related metabolites such as abscisic acid (ABA) and proline. The reduction in excessive accumulation of these compounds suggests a mitigation of drought-induced stress. These beneficial effects are attributed to improved soil water retention and enhanced root–soil interactions facilitated by the porous structure of biochar. The findings highlight the potential of biochar as a climate-resilient input for drought-prone agricultural systems.

The role of biochar in mitigating heavy metal stress is demonstrated in two complementary studies. Qin et al. investigated the effectiveness of *Wedelia trilobata*-derived biochar in alleviating chromium toxicity in Chinese cabbage grown under hydroponic conditions. The study revealed a substantial reduction in chromium accumulation in plant tissues, accompanied by significant improvements in photosynthetic pigments, antioxidant enzyme activities, and nutrient uptake. Furthermore, biochar application reduced oxidative stress markers such as malondialdehyde and hydrogen peroxide, indicating enhanced cellular protection. These results emphasize the strong adsorption capacity of biochar and its effectiveness in immobilizing toxic metals, thereby reducing their bioavailability and phytotoxicity.

Similarly, Khan et al. examined the combined application of biochar and melatonin in mitigating lead toxicity in rice. The co-application strategy significantly reduced lead accumulation in plant tissues and improved growth and physiological performance. Notably, the combined treatment enhanced antioxidant enzyme activities and regulated the expression of genes involved in metal uptake and stress response. This study provides important insights into the integration of biochar with plant growth regulators, demonstrating a novel approach to enhancing stress tolerance at both physiological and molecular levels.

Taken together, the studies in this Research Topic highlight several key insights. First, biochar enhances plant physiological and biochemical responses, particularly by strengthening antioxidant defense systems and regulating osmolyte accumulation. Second, biochar plays a crucial role in immobilizing toxic ions, reducing their uptake and mitigating their harmful effects on plants. Finally, the integration of biochar with other amendments, such as microbial inoculants and signaling molecules, emerges as a highly effective strategy for improving plant resilience under diverse stress conditions.

Despite these promising findings, several research gaps remain. Most studies are conducted under controlled conditions, and there is a need for long-term field-based studies to validate the effectiveness of biochar under diverse agroecological systems. Additionally, the variability in biochar properties due to differences in feedstock and pyrolysis conditions necessitates further standardization and optimization. Future research should also focus on understanding the interactions between biochar, soil microbial communities, and plant signaling pathways to fully exploit its potential in sustainable agriculture.

In conclusion, this Research Topic provides strong evidence that biochar-based approaches offer a viable and sustainable solution for mitigating abiotic stresses in plants. By improving soil quality, enhancing plant physiological resilience, and reducing stress-induced damage, biochar holds significant promise for advancing climate-resilient agriculture. The integration of biochar with complementary strategies further strengthens its applicability, paving the way for innovative and sustainable agricultural practices in the face of growing environmental challenges.
